# Not that different after all: Pro‐environmental social norms predict pro‐environmental behaviour (also) among those believing in conspiracy theories

**DOI:** 10.1111/bjop.70030

**Published:** 2025-09-15

**Authors:** Kevin Winter, Lotte Pummerer, Kai Sassenberg

**Affiliations:** ^1^ University of Hohenheim Stuttgart Germany; ^2^ Bergische Universität Wuppertal Wuppertal Germany; ^3^ University of Bremen Bremen Germany; ^4^ Leibniz‐Institut für Wissensmedien Tübingen Germany; ^5^ Leibniz Institute for Psychology Trier Germany; ^6^ University of Trier Trier Germany

**Keywords:** conspiracist worldview, conspiracy beliefs, pro‐environmental behaviour, social influence, social norms

## Abstract

Social norms are powerful predictors of pro‐environmental behaviour. At the same time, conspiracy beliefs are prevalent that can reduce individuals' efforts to act pro‐environmentally and might impede the influence of social norms. Across three cross‐sectional studies in three countries (Germany, UK, US; total *N* = 1037), we investigated the interplay between different types of social norm perceptions and conspiracy beliefs in predicting everyday pro‐environmental behaviour. Against two out of three hypotheses, we found no evidence that conspiracy beliefs moderated the relationship between perceived social norms and self‐reported pro‐environmental behaviour. Rather, perceiving higher pro‐environmental social (especially subjective and injunctive) norms was associated with more frequent pro‐environmental behaviour – also among those with stronger conspiracy beliefs. Conspiracy beliefs (especially those related to climate change) were, in turn, related to less pro‐environmental behaviour. These findings shed light on the social factors that might influence individuals believing in conspiracy theories and give reason for optimism regarding the possibility to overcome their climate inaction via normative influence.

## BACKGROUND

Substantial portions of many populations around the globe believe in conspiracy theories about climate change. According to a YouGov poll, for instance, 27% of respondents in the US and still 9% in the UK believed that human‐made global warming is a hoax invented to deceive people (Ibbetson, 2021). These numbers are alarming given the high consensus among climate scientists about the fact that climate change is mainly caused by human activity (Cook et al., 2016; Myers et al., 2021). However, there is also a substantial number of people who care about environmental issues, and in many countries, questions about sustainability have become a normative issue (Andre et al., 2024; Leiserowitz et al., 2023; Umweltbundesamt, 2023). Governments, industry and institutions communicate that pro‐environmental behaviours are desirable (i.e., injunctive norms). Also, changes in actual behaviours, for example, in the use of solar panels and renewable energy become visible (i.e., descriptive norms). Additionally, people might perceive an expectation from their friends and family to be more environmentally friendly (i.e., subjective norms). Social norms are quite powerful in that they guide people “without the force of law” (Cialdini et al., 1990) and impact pro‐environmental behaviour even without people being necessarily aware of their influence (Barth et al., 2016; Nolan et al., 2008). But do they also predict pro‐environmental behaviour among individuals higher in conspiracy beliefs?

Investigating specifically this group is worthwhile, as believing in conspiracy theories has been linked to lower pro‐environmental attitudes and intentions (Biddlestone et al., 2022; Winter, Hornsey, et al., 2022). At the same time, individuals believing in conspiracy theories show high levels of reactance (Hornsey et al., 2018b), which might prevent them from following (specific types of) social norms. As of now, research on whether conspiracy beliefs moderate the relationship between social norms and pro‐environmental behaviour is scarce. Also, no research has as of yet examined potential differences in the relationship between different types of conspiracy beliefs and different types of social norms in the environmental domain. Identifying boundary conditions that potentially undermine or strengthen the relationship between social norms and pro‐environmental behaviour is important to prevent and overcome climate inaction.

### Social norms and pro‐environmental behaviour

Social norms are quite a strong predictor of human behaviour, and decades of psychological research have provided support for the effectiveness of social norms as a means to foster behaviour change (Cialdini & Trost, 1998). More specifically, there is strong evidence that social norms can promote pro‐environmental behaviour (Cialdini & Jacobson, 2021; Helferich et al., 2023) and that social identity processes (including social norms) more generally predict both individual and collective pro‐environmental action (Fritsche et al., 2018). One common distinction in the literature on social norms is the one between injunctive and descriptive norms. *Injunctive* norms refer to the perception of what others consider appropriate behaviour and what they expect from the individual, while *descriptive* norms refer to the perception of whether most others actually perform a certain (e.g., pro‐environmental) behaviour (Cialdini et al., 1990; Stok et al., 2014). Although injunctive and descriptive pro‐environmental norms are positively related, both explain independent and equally sized portions of variance in pro‐environmental behaviour (Helferich et al., 2023). This might be due to the distinct motivational bases underlying them. Injunctive norms suggest that (not) performing a certain behaviour could be sanctioned by others and, thus, put social pressure on individuals to act in line with the majority's expectations. Descriptive norms, in turn, provide information on which behaviours are “normal” in a certain reference group and, thus, serve as a cognitive shortcut for adaptive and effective behaviour (Cialdini et al., 1990).

According to the theory of planned behaviour (Ajzen, 1991), there is another type of social norm that is relevant to explain individuals' intentions and behaviours. Here, the *subjective* norm is referred to as the expectation by important others to perform a certain behaviour. Accordingly, injunctive norms stress the perceived societal pressure, whereas subjective norms refer to the expectations of those with presumably good intentions (Cialdini & Jacobson, 2021). The subjective norm and other constructs from the theory of planned behaviour are frequently used to explain pro‐environmental intentions and behaviour (Yuriev et al., 2020). For instance, pro‐environmental subjective norms positively predict individuals' pro‐environmental behaviours and intentions in several contexts including pro‐environmental consumer behaviour (Xu et al., 2022), intentions to buy an electric vehicle (Barth et al., 2016) and pro‐environmental behavioural intentions at the workplace (Greaves et al., 2013). Overall, meta‐analyses show that the subjective norm predicts several types of pro‐environmental behaviours over and above other types of norms (Niemiec et al., 2020). Thus, different types of social norms are important predictors of pro‐environmental behaviour. But is this also the case among individuals high in conspiracy beliefs?

### Belief in conspiracy theories and climate action

Conspiracy theories are explanations for important events that involve secret plots by powerful groups carried out for their own benefit and at the cost of others (Douglas et al., 2017; Sassenberg et al., 2023). Some of these theories suggest that human‐made climate change is a hoax put forward to deceive the people. Others revolve around the seemingly ulterior motives of climate scientists, governments and industry. While potentially, there are also conspiracy theories surrounding climate change alleging too little engagement by powerholders, including politicians (Pummerer et al., 2025), when speaking of climate change conspiracy theories, we hereby mean conspiracy theories that doubt the existence or human contribution to climate change.

A recent meta‐analysis found that believing in such climate change conspiracy theories is related to a range of attitudes and behaviours that potentially hinder effective climate action, including lower support for climate‐friendly policies and lower intentions to show pro‐environmental behaviour (Biddlestone et al., 2022; see also Tam & Chan, 2023). This conclusion mainly relies on correlational evidence. But there is also some support for a causal link between exposition to climate change conspiracy theories and reduced pro‐environmental behaviour. For instance, watching a climate change conspiracy video (compared to a pro‐climate video and a neutral control condition) reduced the likelihood of study participants clicking on a link to sign a pro‐environmental petition (van der Linden, 2015). Similarly, reading a climate change conspiracy article (compared to an anti‐conspiracy article and a neutral control condition) led to lower pro‐environmental intentions in the private sphere (e.g., using energy‐efficient devices, using environmentally friendly means of transport; Jolley & Douglas, 2014).

While the negative link between climate change conspiracy belief and pro‐environmental attitudes and intentions is quite obvious, the picture is much more heterogeneous for the general propensity to believe in conspiracy theories. This general tendency is often referred to as conspiracist ideation (Lewandowsky et al., 2013), conspiracist worldview (Hornsey, 2020) or conspiracy mentality (Imhoff & Bruder, 2014). It relates to the observation that believing in one conspiracy theory is often associated with believing in conspiracy theories in other (unrelated) domains (Swami et al., 2010). There is some evidence that people with a stronger conspiracist worldview are more likely to reject climate science (Lewandowsky et al., 2013; Uscinski & Olivella, 2017), but this relationship seems to hold primarily in the US (Hornsey et al., 2018a). However, more recently, a conspiracist worldview has been identified as an important predictor of opposition to wind farms, also in other countries (Winter et al., 2024, 2025; Winter, Hornsey, et al., 2022). In light of this emerging research strand, it seems worthwhile to further explore the link between a conspiracist worldview and pro‐environmental behaviour.

While both social norms and conspiracy beliefs have been identified as relevant predictors of attitudes and behaviours in the environmental domain, to date they have been mostly examined in isolation. There might be reasons, however, to assume that social norms and conspiracy beliefs interact in predicting pro‐environmental behaviour. More precisely, conspiracy beliefs might moderate the relationship between specific types of social norms and pro‐environmental behaviour in different ways.

### Normative influence and the belief in conspiracy theories

Several strands of the literature give reason to assume that those believing in conspiracy theories are less influenced by social norms. First, conspiracy belief is related to personal characteristics such as a need for uniqueness (Imhoff & Lamberty, 2017; Lantian et al., 2017), narcissism (Stasielowicz, 2022) and reactance (Hornsey et al., 2018b) that are marked by the tendency to not (wanting to) be influenced by others. Second, conspiracy belief predicts various attitudes and behaviours that are seen as non‐normative (e.g., refusing health regulations, engaging in non‐normative collective action or in everyday crimes; for an overview, see Pummerer, 2022). Even some definitions of conspiracy theories refer to them as “going against the mainstream” (Brotherton, 2013). Yet, there is also research indicating that people higher in conspiracy beliefs are not mute to social norms: Pro‐vaccination subjective norms have been shown to be related to higher vaccination intentions – especially among individuals higher in conspiracy beliefs (Winter, Pummerer, et al., 2022). Also, thinking about reasons for social norms increases adherence to them among individuals higher in conspiracy beliefs (Pummerer, Ditrich, et al., 2022), and opinions of a majority (i.e., descriptive norm communication) guide individuals' judgements irrespective of their conspiracy beliefs (Pummerer et al., 2024). However, there is no research to date examining whether conspiracy beliefs moderate the relationship between social norms and pro‐environmental behaviour. Also, previous research has not tested whether different types of norms interact differently with conspiracy beliefs, while there are good theoretical arguments to make this assumption.

The reasoning that conspiracy beliefs are linked to reactance against the majority or influential authorities therein (Hornsey et al., 2018b; Imhoff & Lamberty, 2017) should be particularly relevant to *injunctive* norms. Especially if others in society explicitly formulate the expectation and exert social pressure to perform a certain behaviour, this could be perceived as a threat to one's personal freedom and accordingly cause reactance (Brehm & Brehm, 1981). Additionally, injunctive norms are often communicated or at least supported by authorities such as public institutions or scientists. Given that conspiracy beliefs are linked to suspicion specifically against powerholders (Imhoff & Bruder, 2014; Lamberty & Imhoff, 2018) and distrust in institutions (Pummerer, Böhm, et al., 2022), injunctive norms might be rejected by individuals higher in conspiracy beliefs.

This could be less the case for the *descriptive* societal norm, which might not be perceived as much as a freedom threat and less connected to authorities, because it less directly conveys expectations and presumably exerts less social pressure. Recent research actually showed that people generally followed the descriptive societal norm in a hypothetical scenario – irrespective of their conspiracist worldview (Pummerer et al., 2024). This suggests that believing in conspiracy theories is not necessarily related to less adherence to descriptive societal norms.

The same might be true for the *subjective* norm. Theoretically, what important others think about pro‐environmental behaviour should also matter for those who believe in conspiracy theories. Believing in conspiracy theories is related to feelings of uncertainty and powerlessness (Jolley & Douglas, 2014; Liekefett et al., 2023). This might be buffered by a good social network or group membership, making norms communicated in such a network or group an important guideline. This seems plausible given that people turn to their group identities when they experience low personal control in order to retain a sense of agency and empowerment (e.g., Fritsche, 2022; Molix & Bettencourt, 2010). For instance, research shows that experimentally inducing a lack of personal control makes people think and act more in line with their ingroup norms (Fritsche et al., 2013). Also, conspiracy belief is related to defending a positive image of oneself and one's ingroup as well as a sense of superiority over other groups (Cichocka et al., 2016). This could mean that the expectations of other ingroup members (e.g., friends and family) are particularly relevant for those high in conspiracy beliefs. In line with this reasoning, perceiving a pro‐vaccination subjective norm (i.e., that important others expect one to get vaccinated) was even more predictive of vaccination intentions among those with a stronger conspiracist worldview (Winter, Pummerer, et al., 2022). Similarly, a pro‐environmental subjective norm could be especially predictive for pro‐environmental behaviour among those with pronounced conspiracy beliefs. Taken together, we hypothesize the following pattern of results for the respective norm types.^1^

The positive relationship of the perceived pro‐environmental injunctive norm and pro‐environmental behaviour is weaker among those with stronger conspiracy beliefs.

The positive relationship of the perceived pro‐environmental descriptive norm and pro‐environmental behaviour is independent of conspiracy beliefs.

The positive relationship of the perceived pro‐environmental subjective norm and pro‐environmental behaviour is stronger among those with stronger conspiracy beliefs.

### The current research

The current work examines the interplay between social norms and conspiracy beliefs with regard to pro‐environmental behaviour in three cross‐sectional studies conducted in three countries (Study 1: US, Study 2: Germany, Study 3: UK). These countries were chosen based on availability rather than theoretical considerations, and it might be that different effects of social norms would occur in other (e.g., more collectivistic) countries (Eom et al., 2016; Sherman et al., 2022). This and the potential lack of generalizability notwithstanding (see also General Discussion), we aimed for a first test of our hypotheses in samples that were relatively easy to reach. In all studies, we assessed climate change conspiracy beliefs, a conspiracist worldview and pro‐environmental behaviour. In Studies 1 and 2, we included measures of all norm types (i.e., subjective, injunctive, descriptive) and, thus, were able to test [Link], [Link], [Link]. In Study 3, we focused on the role of the subjective norm, testing H3 only. Conspiracist worldview was assessed either as agreement with several specific conspiracy theories (Study 1) or as conspiracy mentality (Studies 2 and 3). Studies 2 and 3 were preregistered (Study 2: https://aspredicted.org/rkdx‐2mfs.pdf, Study 3: https://aspredicted.org/rtng‐g7xg.pdf). To adequately test H2 (i.e., the absence of an interaction), we ran Bayesian regression analyses in addition to the preregistered multiple linear regressions to be able to assess the evidence in favour of the null hypothesis. For the sake of consistency, we report Bayes factors for all hypothesis tests throughout the manuscript. All Bayes factors result from a comparison of the model including the interaction effect with a model including only the two main effects, respectively. Thus, BF_10_ indicates the likelihood of the observed data under a model including both main effects and the interaction compared to a model only including the two main effects. Bayesian analyses were conducted with the software JASP (0.19.3.0), while the remaining analyses were carried out using SPSS v29.

Data and code are available via PsychArchives (data: https://doi.org/10.23668/psycharchives.21209, code: https://doi.org/10.23668/psycharchives.21210). The studies received ethics approval from the University of Hohenheim (#2024/44_Winter) and were conducted in line with the ethical guidelines of the American Psychological Association.

## STUDY 1

### Materials and methods

#### Participants and design

Study 1 was a cross‐sectional survey distributed on the platform Amazon Mechanical Turk (MTurk) in March 2021. Participants received 1.50 US$ for filling out a larger package of questionnaires of which the current materials were part. For the sake of consistency and given the lack of a preregistration for Study 1, we applied the criteria of exclusion that were preregistered for Studies 2 and 3. For Study 1, no a priori power analysis was carried out. Three hundred US citizens completed our survey, of which 58 were excluded (partly overlapping: *n* = 57 for failing our attention check item, *n* = 3 who indicated to have completed the survey multiple times). Thus, our final sample consisted of *N* = 242 participants (age: *M* = 39.72, *SD* = 11.89; gender: 84 female, 158 male). With a sample of this size, we would be able to detect a small effect (*f* ^2^ = .03) testing for *R* ^2^ increase of a single regression coefficient in a linear multiple regression analysis with three predictors, (1–*β*) = .80, *α* = .05.

#### Measures and procedure

After giving informed consent, participants were asked the following questions in the indicated order. Participants were presented with a list of the same 16 behaviours regarding which they were asked to report injunctive, descriptive and subjective norms, as well as their own performance of these behaviours. The order of type of norms and behaviours within the norm type was randomized within the lists. The items of interest were four pro‐environmental behaviours (i.e., “use public transport in order to reduce the environmental impact”, “reduce one's meat intake for environmental reasons”, “reduce one's flights in order to protect the environment”, “take the car only when necessary in order to reduce emissions”, complete list of items in the Appendix S1). We focused here on mobility and food as these are among the most important aspects of private‐sphere pro‐environmental behaviour (e.g., Hopwood et al., 2023; Kaiser & Wilson, 2004).

To assess the perceived pro‐environmental *injunctive norm*, participants were instructed to estimate “how many people in society say one should…” on a scale from 0% to 100% with regard to the abovementioned behaviours. For the *descriptive norm*, we asked participants to estimate “how many people in society are doing the described behaviors” and for the *subjective norm* “how many of the people you care about say one should…”. Please note, that it is more common in the literature to use a personalized form (“you/I should”) to assess the subjective norm (e.g., Greaves et al., 2013; Xu et al., 2022). However, more general forms as the one we used are also prevalent (e.g., Barth et al., 2016; White et al., 2009). The core feature of a subjective norm across the literature is that it refers to a specific group, namely “important others” (see also Cialdini & Jacobson, 2021).

Next, their own *self‐reported pro‐environmental behaviour* was assessed with regard to the same behaviours following the instruction: “You will see the behaviors one last time. Now we ask you, whether you display the behaviors. Please indicate your agreement/disagreement with the following sentences” (on a scale from 1 = *totally disagree* to 11 = *totally agree*; e.g., “I use public transport in order to reduce the environmental impact”).

*Belief in climate change conspiracy theories* was assessed with four items (one from Jolley & Douglas, 2014; e.g., “Climate change is a hoax” on a scale from 1 = *strongly disagree* to 7 = *strongly agree*). Participants' *conspiracist worldview* was measured with their agreement to a set of six specific conspiracy theories (e.g., “The Apollo moon landings never happened and were staged in a Hollywood film studio”; Lewandowsky et al., 2013) on the same response scale. After completing these measures, participants indicated demographic data and received their financial compensation. Descriptive statistics, intercorrelations and internal consistencies of the measures are presented in Table 1.

**TABLE 1:** Descriptive statistics, intercorrelations and internal consistencies (McDonald's *ω*) of all measures (Study 1; *N* = 242).

	Subjective norm	Injunctive norm	Descriptive norm	Self‐reported pro‐environmental behaviour	Climate change conspiracy belief	Conspiracist worldview
	(1)	(2)	(3)	(4)	(5)	(6)
(1)	*ω* = .93	.71***	.69***	.71***	−.23***	.17**
(2)		*ω* = .92	.65***	.58***	−.15*	.17**
(3)			*ω* = .93	.68***	.13*	.46***
(4)				*ω* = .86	−.15*	.29***
(5)					*ω* = .87	.50***
(6)						*ω* = .93
*M* (*SD*)	42.02 (27.65)	45.65 (24.18)	33.86 (23.63)	5.33 (3.10)	2.41 (1.46)	3.07 (1.79)
Scale range	0–100	0–100	0–100	1–11	1–7	1–7

*p < .05, **p < .01, **p < .001.

#### Analysis plan

We ran one linear multiple regression analysis for each combination of norm type and indicator of conspiracy belief (i.e., resulting in six analyses for Study 1). Perceived norm and conspiracy belief were mean‐centred before entering them into the regression. Together with the norm × conspiracy belief interaction, they were included as predictors of self‐reported pro‐environmental behaviour. In line with the preregistrations of Studies 2 and 3, we excluded participants as statistical outliers if their absolute studentized deleted residuals (SDR) in the respective analysis were larger than 2.59 (based on the recommendations of Neter et al., 1996); that is, if the likelihood of their responses' deviation from the regression line (i.e., the residual) is below 1%. This explains the slightly different degrees of freedom per analysis. Given that each hypothesis was tested twice (i.e., for both types of conspiracy belief), we adjusted the significance level to *α* = .025 (Bonferroni correction).

### Results

#### Conspiracy beliefs and injunctive norm

A significant interaction of conspiracy belief and injunctive norm emerged for both climate change conspiracy belief, *β* = .16, *p* = .004, BF_10_ = 6.12, and conspiracist worldview, *β* = .13, *p* = .006, BF_10_ = 4.40. However, the direction of the interaction was opposite to what we predicted in H1. That is, the positive link between perceived pro‐environmental injunctive norm and self‐reported pro‐environmental behaviour was actually *stronger* when conspiracy beliefs were high (i.e., +1*SD*), *β* = .74 to.77, both *p*s < .001, compared to low (i.e., −1*SD*), both *β*s = .49, both *p*s < .001. The full results of the regression analyses testing H1 are presented in Table 2.

**TABLE 2:** Results of multiple linear regression analyses testing H1 (injunctive norm; IN) across studies with different indicators of conspiracy belief.

	Study 1						Study 2					
	*B* (*SE*)	95% CI	*β*	*t*	*df*	*p*	*B* (*SE*)	95% CI	*β*	*t*	*df*	*p*
Climate change conspiracy belief (CCB)												
CCB	−0.0001 (0.12)	−0.23, 0.23	−.0001	−0.001	234	.999	−0.11 (0.02)	−0.15, −0.08	−.37	−6.05	251	<.001
IN	0.08 (0.01)	0.07, 0.09	.63	12.82	234	<.001	0.004 (0.002)	0.0002, 0.01	.12	2.09	251	.038
CCB × IN	0.01 (0.004)	0.004, 0.02	.16	2.88	234	.004	−0.001 (0.001)	−0.003, 0.002	−.03	−0.47	251	.638
Conspiracist worldview (CW)												
CW	0.29 (0.08)	0.12, 0.45	.17	3.45	233	<.001	−0.06 (0.02)	−0.09, −0.02	−.20	−3.20	252	.002
IN	0.08 (0.01)	0.07, 0.09	.62	12.80	233	<.001	0.01 (0.002)	0.002, 0.01	.18	2.97	252	.003
CW × IN	0.10 (0.003)	0.003, 0.02	.13	2.78	233	.006	−0.001 (0.001)	−0.004, 0.001	−.06	−1.03	252	.306

*Note*: Different degrees of freedom within studies result from outlier exclusions.

#### Conspiracy beliefs and descriptive norm

Multiple linear regressions yielded no evidence for an interaction between descriptive norm perception and either climate change conspiracy belief, *β* = −.04, *p* = .465, BF_10_ = 0.11, or conspiracist worldview, *β* = .04, *p* = .417, BF_10_ = 0.13. The Bayesian analyses revealed that there was substantial evidence in favour of the null hypothesis (according to Kass & Raftery, 1995; see also Wagenmakers, 2007). The data were more than eight times more likely to be observed under the model including only the two main effects but not the interaction. These results, thus, provide support for H2. Climate change conspiracy belief negatively predicted self‐reported pro‐environmental behaviour, *β* = −.28, *p* < .001, but conspiracist worldview did not, *β* = −.03, *p* = .581. The main effects of the perceived pro‐environmental descriptive norm were significant in both analyses, *β*s = .74–.78, both *p*s < .001. The full results of the regression analyses testing H2 are presented in Table 3.

**TABLE 3:** Results of multiple linear regression analyses testing H2 (descriptive norm; DN) across studies with different indicators of conspiracy belief.

	Study 1						Study 2					
	*B* (*SE*)	95% CI	*β*	*t*	*df*	*p*	*B* (*SE*)	95% CI	*β*	*t*	*df*	*p*
Climate change conspiracy belief (CCB)												
CCB	−0.59 (0.10)	−0.79, −0.40	−.28	−6.00	232	<.001	−0.12 (0.02)	−0.16, −0.08	−.38	−6.46	251	<.001
DN	0.10 (0.01)	0.09, 0.11	.78	17.82	232	<.001	0.002 (0.002)	−0.002, 0.005	.05	0.75	251	.451
CCB × DN	−0.003 (0.01)	−0.01, 0.01	−.04	−0.73	232	.465	−0.0004 (0.001)	−0.003, 0.002	−.02	−0.25	251	.801
Conspiracist worldview (CW)												
CW	−0.05 (0.09)	−0.22, 0.12	−.03	−0.55	232	.581	−0.05 (0.02)	−0.09, −0.02	−.19	−3.00	250	.003
DN	0.10 (0.01)	0.08, 0.11	.74	14.29	232	<.001	0.001 (0.002)	−0.003, 0.01	.04	0.58	250	.565
CW × DN	0.003 (0.003)	−0.004, 0.01	.04	0.81	232	.417	−0.001 (0.002)	−0.01, 0.002	−.06	−0.93	250	.354

*Note*: Different degrees of freedom within studies result from outlier exclusions.

#### Conspiracy beliefs and subjective norm

The interaction between conspiracy beliefs and pro‐environmental subjective norm was significant for both climate change conspiracy belief, *β* = .24, *p* < .001, BF_10_ = 17,013.54, and conspiracist worldview, *β* = .18, *p* < .001, BF_10_ = 627.87. In both cases and in line with H3, the subjective norm predicted pro‐environmental behaviour to a stronger degree when conspiracy belief was high, *β* = .92 to.98, both *p*s < .001, compared to when it was low, *β* = .54 to .57, both *p*s < .001. Full results of the regression analyses testing H3 are presented in Table 4.

**TABLE 4:** Results of multiple linear regression analyses testing H3 (subjective norm; SN) across studies with different indicators of conspiracy belief.

	Study 1						Study 2						Study 3					
	*B* (*SE*)	95% CI	*β*	*t*	*df*	*p*	*B* (*SE*)	95% CI	*β*	*t*	*df*	*p*	*B* (*SE*)	95% CI	*β*	*t*	*df*	*p*
Climate change conspiracy belief (CCB)																		
CCB	0.27 (0.10)	0.07, 0.46	.13	2.70	235	.008	−0.08 (0.02)	−0.12, −0.05	−.25	−4.92	253	<.001	−0.06 (0.01)	−0.08, −0.04	−.22	−5.78	519	<.001
SN	0.09 (0.01)	0.08, 0.09	.76	18.05	235	<.001	0.01 (0.001)	0.01, 0.02	.48	9.29	253	<.001	0.01 (0.001)	0.01, 0.013	.46	12.13	519	<.001
CCB × SN	0.02 (0.003)	0.01, 0.02	.24	5.10	235	<.001	0.002 (0.001)	0.0001, 0.004	.10	2.04	253	.042	−0.0002 (0.001)	−0.001, 0.001	−.02	−0.40	519	.692
Conspiracist worldview (CW)																		
CW	0.27 (0.07)	0.13, 0.41	.16	3.78	235	<.001	−0.04 (0.02)	−0.08, −0.01	−.15	−2.83	254	.005	−0.03 (0.01)	−0.06, −0.01	−.12	−3.03	522	.003
SN	0.08 (0.01)	0.07, 0.09	.74	18.02	235	<.001	0.01 (0.001)	0.01, 0.02	.50	9.50	254	<.001	0.01 (0.001)	0.01, 0.014	.48	12.63	522	<.001
CW × SN	0.01 (0.003)	0.01, 0.02	.18	4.34	235	<.001	−0.001 (0.001)	−0.003, 0.001	−.04	−0.77	254	.444	−0.001 (0.001)	−0.002, 0.001	−.04	−0.91	522	.364

*Note*: Different degrees of freedom within studies result from outlier exclusions.

### Discussion

The results of the current study suggest that also those believing in conspiracy theories act in line with the pro‐environmental norms they perceive. Contrary to H1, this was even the case for a pro‐environmental injunctive norm; in other words, when individuals perceived a strong societal expectation to act pro‐environmentally. We hypothesized that this norm would be *less* predictive of pro‐environmental behaviour among those with stronger conspiracy beliefs. However, the opposite was the case: the relationship between injunctive norms and pro‐environmental behaviour was even *stronger* among those with stronger conspiracy beliefs. In any case, this finding suggests that those higher in conspiracy beliefs do not necessarily go against the expectations of society. In line with H2, our findings suggest that those high in conspiracy beliefs are as receptive to the descriptive (societal) norm when it comes to pro‐environmental behaviour. This resembles recent studies demonstrating an effect of majority opinions on those with stronger conspiracy beliefs (Pummerer et al., 2024). We also found support for H3, that the perceived pro‐environmental subjective norm (i.e., the expectations of important others) predicted pro‐environmental behaviour to an even stronger degree when conspiracy beliefs were high (rather than low). This is in line with previous findings on the role of pro‐vaccination subjective norms (Winter, Pummerer, et al., 2022). Notably, with regard to the effects of descriptive and subjective norms among those with a strong conspiracist worldview found earlier, we extended these findings showing similar effects among those believing in a context‐specific (climate change) conspiracy theory.

While the interaction patterns were similar irrespective of the type of conspiracy beliefs included in the analyses, it is worth mentioning that the bivariate correlations between these types of conspiracy beliefs on the one hand and pro‐environmental behaviour on the other hand looked quite distinct. As one would expect based on previous findings (Biddlestone et al., 2022), climate change conspiracy belief was negatively related to self‐reported pro‐environmental behaviour. However, the correlation between conspiracist worldview and pro‐environmental behaviour was positive – which is contrary to previous findings in this domain (Lewandowsky et al., 2013; Winter, Hornsey, et al., 2022). Notably, this positive relationship was gone when analysing conspiracist worldview together with the descriptive norm in a regression (testing H2). This might indicate that the positive relationship between conspiracist worldview and pro‐environmental behaviour could be fully explained by the more positive descriptive norm perception found among those with a stronger conspiracist worldview.

Although surprising results are not necessarily an indicator of low data quality and although we already excluded participants who failed our attention check, there are general concerns about data quality on MTurk (Chmielewski & Kucker, 2020; Douglas et al., 2023). Thus, one goal of Study 2 was to test the robustness of the associations with conspiracist worldview using another measure and shifting to another recruiting platform. Generally, we aimed to conceptually replicate the findings of Study 1 with slightly adapted measures and in a different country (i.e., Germany).

## STUDY 2

### Materials and methods

#### Participants and design

In March 2024, we conducted a cross‐sectional survey on the platform Prolific Academic, which tends to provide higher data quality than MTurk (Douglas et al., 2023). Participants received a compensation of 1.20£. Based on an a‐priori power analysis for *R* ^2^ increase in a multiple regression analysis, a sample size of *N* = 264 would be sufficient to detect a small effect (*f* ^2^ = .03) with (1 – *β*) = .80 and *α* = .05 (one predictor tested, three predictors in total). Two hundred seventy‐three German adults completed our survey. Two of them withdrew their participation at the end of the survey. Of the remaining sample, 10 participants were excluded based on our preregistered criteria (*n* = 8 for indicating to have participated multiple times; *n* = 2 who indicated to not speak German fluently; see https://aspredicted.org/rkdx‐2mfs.pdf). The final sample consisted of *N* = 261 participants (age: *M* = 31.39, *SD* = 9.88; gender: 126 female, 130 male, 4 other, 1 did not want to report).

#### Measures and procedure

The measures and procedure were similar to Study 1 with some adaptations. After giving informed consent, participants first reported on their own *pro‐environmental behaviour*. This change in order also served to prevent that participants' responses were guided by their responses on the social norm questions and, thus, to reduce an overestimation of the actual intercorrelations. The scale contained 15 items that covered a broader range of behavioural domains than the one used in Study 1 (e.g., “When it gets cold in my flat, I put on something warmer instead of turning up the heating”, “I buy seasonal fruits and vegetables”; Wullenkord & Reese, 2021). Participants responded on a 5‐point scale (1 = *never*, 2 = *rarely*, 3 = *sometimes*, 4 = *often*, 5 = *always*).

Then we assessed the perceived *descriptive norm* for the same behaviours asking “Please estimate: What percentage of people in Germany often or always show the following behaviors?”. Participants responded on a scale from 0% to 100%. The statement “Now it's not about the actual behavior of others, but about what they think one should do” preceded the assessment of the *injunctive norm*. Participants rated every behaviour from 0% to 100% following the instruction “Please estimate: What percentage of people in Germany say one should…?”. Then, the *subjective norm* for each behaviour was assessed from 0% to 100% after the instruction “Now it's no longer about the people in Germany, but about what the people who are important to you personally think. Please estimate: What percentage of the people who are important to you say that one should…?”

Afterwards, *belief in climate change conspiracy theories* was assessed with five items (e.g., “The idea that the world is headed for catastrophic climate change is a fraud”; Jolley & Douglas, 2014) on a scale from 1 = *do not agree at all* to 7 = *fully agree*. Finally, we assessed conspiracist worldview with a different measure than in Study 1, namely the Conspiracy Mentality Questionnaire (Bruder et al., 2013). This scale does not ask for agreement with several specific conspiracy theories but captures the generalized tendency to perceive societal events as determined by evil plots of malevolent groups (e.g., “Many important things in the world happen without the public being informed”; from 1 = *do not agree at all* to 7 = *fully agree*). Participants were paid after indicating their demographics. For descriptive statistics, internal consistencies and intercorrelations of the measures, see Table 5.

**TABLE 5:** Descriptive statistics, intercorrelations and internal consistencies (McDonald's *ω*) of all measures (Study 2; *N* = 261).

	Subjective norm	Injunctive norm	Descriptive norm	Self‐reported pro‐environmental behaviour	Climate change conspiracy belief	Conspiracist worldview
	(1)	(2)	(3)	(4)	(5)	(6)
(1)	*ω* = .87	.53***	.20**	.50***	−.17**	−.08
(2)		*ω* = .89	.41***	.22***	−.17**	−.05
(3)			*ω* = .85	.05	.07	.12
(4)				*ω* = .68	−.31***	−.17**
(5)					*ω* = .93	.58***
(6)						*ω* = .90
*M* (*SD*)	58.71 (16.00)	62.56 (14.14)	44.01 (11.73)	3.48 (0.43)	2.00 (1.31)	3.79 (1.43)
Scale range	0–100	0–100	0–100	1–5	1–7	1–7

*p < .05, **p < .01, **p < .001.

### Results

As in Study 1, we conducted two analyses per hypothesis (i.e., one for each type of conspiracy belief, respectively) resulting in a total of six multiple regression analyses. Full results including all relevant statistics are presented in Tables 2, 3, 4.

#### Conspiracy beliefs and injunctive norm

Contrary to H1, there was no significant interaction between either climate change conspiracy belief and the perceived pro‐environmental injunctive norm, *β* = −.03, *p* = .638, BF_10_ = 0.20, or conspiracist worldview and injunctive norm, *β* = −.06, *p* = .306, BF_10_ = 0.36. Higher climate change conspiracy belief, *β* = −.37, *p* < .001, and higher conspiracist worldview, *β* = −.20, *p* = .002, predicted less self‐reported pro‐environmental behaviour. In the regression with climate change conspiracy belief, the injunctive norm and their interaction as predictors, the main effect of the perceived injunctive norm was not significant (after Bonferroni correction), *β* = .12, *p* = .038. However, a stronger perceived injunctive norm significantly predicted more self‐reported pro‐environmental behaviour in the regression analysis with conspiracist worldview, injunctive norm and their interaction as predictors, *β* = .18, *p* = .003.

#### Conspiracy beliefs and descriptive norm

The preregistered multiple linear regressions revealed no evidence for an interaction between descriptive norm perception and either climate change conspiracy belief, *β* = −.02, *p* = .801, BF_10_ = 0.19 (BF_10_ = 0.04 when compared to a model only including the significant main effect of climate change conspiracy beliefs), or conspiracist worldview, *β* = −.06, *p* = .354, BF_10_ = 0.36 (BF_10_ = 0.08 when compared to a model only including the significant main effect of conspiracist worldview). This means that the observed data were (depending on the type of conspiracy belief) about three to five times more likely under the main effects compared to the interaction model. Thus, Study 2 provides additional support for the absence of an interaction between descriptive norms and conspiracy beliefs (i.e., supporting H2). As noted above, climate change conspiracy belief negatively predicted self‐reported pro‐environmental behaviour, *β* = −.38, *p* < .001, as did conspiracist worldview, *β* = −.19, *p* = .003. There was no evidence for a main effect of the perceived descriptive norm in either of the analyses, *β*s = .04 to.05, *p*s > .450.

#### Conspiracy beliefs and subjective norm

Contrary to H3, we found no evidence for an interaction between subjective norm and climate change conspiracy belief (after Bonferroni correction), *β* = .10, *p* = .042, BF_10_ = 0.93. But if anything, the positive correlation between pro‐environmental subjective norm and pro‐environmental behaviour was stronger among those with high climate change conspiracy belief (+1*SD*), *β* = .58, *p* < .001, than among those with low climate change conspiracy belief (−1*SD*), *β* = .37, *p* < .001. Likewise, no interaction occurred for subjective norm and conspiracist worldview, *β* = −.04, *p* = .444, BF_10_ = 0.18. As in the preceding analyses, climate change conspiracy belief negatively predicted pro‐environmental behaviour, *β* = −.25, *p* < .001, as did conspiracist worldview, *β* = −.15, *p* = .005. The perceived subjective norm positively predicted pro‐environmental behaviour in both the analyses, *β* = .48 to.50, both *p*s < .001.

### Discussion

Other than predicted, conspiracy beliefs did not moderate the relationship between the perceived injunctive norm and pro‐environmental behaviour (H1). Rather, the injunctive norm and both types of conspiracy beliefs predicted self‐reported pro‐environmental behaviour independently (in different directions). It should be noted, however, that the effect of the injunctive norm in the regression including climate change conspiracy beliefs was not significant after Bonferroni correction. For the descriptive norm perception, we expected its influence to be independent of conspiracy beliefs (H2). In line with this prediction, there was no evidence for an interaction between conspiracy beliefs and descriptive norm perception, and Bayesian analyses revealed substantial evidence in favour of the null hypothesis. Interestingly, however, the perceived descriptive norm did not predict pro‐environmental behaviour at all in Study 2. This is somewhat surprising given the pivotal role that injunctive and descriptive norms usually play for pro‐environmental behaviour (Cialdini & Jacobson, 2021; Helferich et al., 2023). There was no evidence for H3 that a pro‐environmental subjective norm was even more predictive of pro‐environmental behaviour among participants with higher conspiracy beliefs. Instead, there was evidence for independent main effects of the subjective norm and both kinds of conspiracy beliefs.

Taken together, Study 2 provides evidence that perceiving a strong pro‐environmental (injunctive and subjective, but not descriptive) norm relates to more pro‐environmental behaviour among participants – irrespective of their conspiracy beliefs. Those conspiracy beliefs (both context‐specific and general) were, in turn, negatively related to self‐reported pro‐environmental behaviour. Studies 1 and 2 together provide a clear picture with regard to H1 and H2: (1) there was no evidence for the predicted interaction between injunctive norms and conspiracy beliefs and (2) there was substantial evidence for the predicted absence of an interaction between descriptive norms and conspiracy beliefs. With regard to H3, the results were mixed: Study 1 provided strong evidence in favour of the predicted interaction between subjective norms and conspiracy beliefs, while there was no evidence for the interaction in Study 2. To provide more clarity with regard to H3, we focused on the role of subjective norms in Study 3, conducting a study in a third country (i.e., the UK). As a further methodological improvement, we timely separated the measurement of predictors and outcome.

## STUDY 3

### Materials and methods

#### Participants and design

Study 3 was again conducted via Prolific Academic in August 2024. This time we recruited UK citizens for a two‐part study on “Society and personal behavior”. An a‐priori power analysis for *R* ^2^ increase in a linear multiple regression analysis indicated that in order to detect a small effect of one out of three predictors (*f* ^2^ = .016) with (1−*β*) = .80 and *α* = .05, we would need a sample size of *N* = 493. To account for drop‐out between the two data collections (which were 1 week apart), our aim was to recruit *N* = 600 participants at T1. The initial sample was quota‐based for age, gender and political party affiliation of the UK population. In total, 606 individuals took part at T1 of which 6 failed an attention check and were, thus, excluded as preregistered (see https://aspredicted.org/rtng‐g7xg.pdf). The remaining 600 participants were reinvited at T2 and 537 actually took part. Two of them were excluded for a failed attention check, and one participant could not be matched to data at T1, which left us with a final sample of *N* = 534 (age: *M* = 47.36, *SD* = 15.54; gender: 279 female, 253 male, 2 did not want to report). Participants received 1.00£ for the completion of both study parts (i.e., 0.60£ for T1, 0.40£ for T2).

#### Measures and procedure

The study was split into two parts participants responded to with a delay of 1 week. This procedure was chosen to reduce a potential overestimation of correlations between the subjective norm rating and the self‐reported pro‐environmental behaviour, which both asked for a judgement on the same set of behaviours. Thus, we assessed all predictor variables at T1 and only the dependent variable at T2. We assessed the perceived *subjective norm* with 15 items as in Study 2. This time, *climate change conspiracy belief* was assessed with the full 7‐item scale by Jolley and Douglas (2014). As in Study 2, we assessed *conspiracist worldview* with the 5‐item Conspiracy Mentality Questionnaire (Bruder et al., 2013). *Self‐reported pro‐environmental behaviour* was measured with the same 15 behaviours as in Study 2. We included four filler items in the pro‐environmental behaviour scale that asked for everyday behaviours not related to the environment (e.g., “I go to the doctor when I'm not feeling well”) to cover the actual purpose of the study. Descriptive statistics, internal consistencies and intercorrelations of the measures are presented in Table 6.

**TABLE 6:** Descriptive statistics, intercorrelations and internal consistencies (McDonald's *ω*) of all measures (Study 3; *N* = 534).

	Subjective norm	Self‐reported pro‐environmental behaviour	Climate change conspiracy belief	Conspiracist worldview
	(1)	(2)	(3)	(4)
(1)	*ω* = .87	.43***	−.13**	−.002
(2)		*ω* = .69	−.24***	−.10*
(3)			*ω* = .96	.53***
(4)				*ω* = .91
*M* (*SD*)	45.47 (16.78)	3.30 (0.44)	2.17 (1.53)	4.25 (1.45)
Scale range	0–100	1–5	1–7	1–7

*p < .05, **p < .01, **p < .001.

### Results

#### Conspiracy beliefs and subjective norm

Following the same procedure as in the previous studies, we conducted two linear multiple regression analyses to test H3 (i.e., one for each type of conspiracy belief, respectively; see Table 4). Against H3, there was no evidence for an interaction between the perceived subjective norm and either climate change conspiracy belief, *β* = −.02, *p* = .692, BF_10_ = 0.11, or conspiracist worldview, *β* = −.04, *p* = .364, BF_10_ = 0.16. There were main effects of both climate change conspiracy belief, *β* = −.22, *p* < .001, and conspiracist worldview, *β* = −.12, *p* = .003. The subjective norm positively predicted self‐reported pro‐environmental behaviours in the analyses with climate change conspiracy belief, *β* = .46, *p* < .001, and conspiracist worldview, *β* = .48, *p* < .001, as predictors, respectively.

### Discussion

The results of Study 3 did not confirm H3 that the perceived subjective norm would even more strongly predict self‐reported pro‐environmental behaviour among those with strong conspiracy beliefs. Instead, both climate change‐specific and general conspiracy beliefs (negatively), and pro‐environmental subjective norm (positively) predicted self‐reported pro‐environmental behaviour independently of each other. Given the methodological improvements of Study 3 and the results of the preceding studies, we deem it appropriate to reject H3 based on the collected data. The current results suggest that perceiving a pro‐environmental subjective norm shapes the behaviour of individuals even if their conspiracy beliefs are high (but apparently not to a stronger degree).

## GENERAL DISCUSSION

The current studies revealed new insights in the interplay between social norms and conspiracy beliefs in predicting pro‐environmental behaviour. Contrary to H1, we found no evidence that the perceived injunctive norm (i.e., expectations of others in society) predicted pro‐environmental behaviour to a lesser degree among those with strong conspiracy beliefs. Rather, injunctive norms predicted pro‐environmental behaviour irrespective of conspiracy beliefs. In line with H2, we found substantial evidence for the absence of an interaction between the perceived descriptive norm (i.e., actual behaviour of others in society) and conspiracy beliefs. That is, the positive relationship between descriptive norms and pro‐environmental behaviour (which was only present in Study 1, but not Study 2) was not moderated by conspiracy beliefs. With regard to H3, a slightly less consistent picture emerged. There was some evidence for the perceived subjective norm (i.e., expectations of important others) being more strongly related to pro‐environmental behaviour among those with strong conspiracy beliefs (Study 1). However, Studies 2 and 3 did not confirm this interaction, but rather provided evidence for its absence. As for injunctive norms, it seems that subjective norms positively predict pro‐environmental behaviour irrespective of conspiracy beliefs. Across studies, climate change conspiracy beliefs were negatively related to pro‐environmental behaviour. Overall, the current studies give little reason to assume that perceived social norms would relate differently to pro‐environmental behaviour depending on individuals' level of conspiracy beliefs.

### Implications for research on social influence

The current research has broader implications for theorizing on social influence and particularly with regard to the role of conspiracy beliefs therein. First, our results are in line with the Theory of Normative Conduct (Cialdini et al., 1990) in that injunctive norms are important predictors of individuals' behaviour. Notably, the theory also highlights the need to differentiate injunctive from descriptive norms. Indeed, although both types of norms were positively related throughout our studies, their relationships with pro‐environmental behaviour looked somewhat different. Actually, descriptive norm perceptions only predicted pro‐environmental behaviour in Study 1, but not Study 2. This finding is surprising given meta‐analytical evidence that both injunctive and descriptive norms explain equal shares of pro‐environmental behaviour (Helferich et al., 2023). Obvious differences between our studies concern the nationality of the samples (Study 1: US, Study 2: Germany) and different measures of pro‐environmental behaviour (Study 1: mostly high‐cost mobility and food‐related items; Study 2: a broader set of low and high‐cost behaviours). Interestingly, the meta‐analysis mentioned above did not find evidence for moderation of these effects by type of pro‐environmental behaviour (low vs. high cost) or cultural influences (samples from mainly collectivistic vs. individualistic cultures; Helferich et al., 2023). Accordingly, future research should more closely investigate the boundary conditions under which the influence of injunctive or descriptive norms might be more or less pronounced. Speaking to this issue, our studies suggest that conspiracy beliefs are unlikely candidates for such a moderator.

Second, our studies confirm that subjective norms as derived from the Theory of Planned Behaviour (Ajzen, 1991) are important predictors of pro‐environmental behaviour (e.g., Yuriev et al., 2020). Indeed, if important others expect one to perform pro‐environmental behaviour, this might be more relevant to individuals' choices than the perceived expectations of broader society. Throughout our studies, subjective norms were the norm type most strongly associated with pro‐environmental behaviour. Accordingly, theorizing on the influence of different norm types in the environmental domain should more clearly separate subjective from injunctive norms – which are often treated interchangeably (Helferich et al., 2023; Niemiec et al., 2020). Notably, we did not take into account further constructs of the theory of planned behaviour such as attitudes towards the behaviour or perceived behavioural control, because we focused on the role of different social norms. However, one might reason that (climate change) conspiracy beliefs reflected a default negative attitude towards pro‐environmental behaviour. Accordingly, it is in line with the Theory of Planned Behaviour that subjective norm and the attitudinal component independently predicted pro‐environmental behaviour.

Third, despite their negative relationship with pro‐environmental behaviour, there was no evidence in our studies that conspiracy beliefs were an obstacle to social influence in the environmental domain. This might be surprising given the general perception of conspiracy believers as being resistant to social influence and going against the mainstream (e.g., Brotherton, 2013; Sunstein & Vermeule, 2009). However, the impression that conspiracy believers might resist social norms results from mainly correlational findings linking conspiracy belief with certain personality variables such as trait reactance (Hornsey et al., 2018b) or need for uniqueness (Imhoff & Lamberty, 2017; Lantian et al., 2017) that are related to reduced social influence (e.g., Dillard & Shen, 2005; Imhoff & Erb, 2009). Studies directly testing the interplay between conspiracy beliefs and (perceived) social norms, yet, paint a more optimistic picture. This research showed that descriptive norms had an influence among individuals high in conspiracy beliefs (Pummerer et al., 2024) and that pro‐vaccination subjective norms buffered the relationship between conspiracy beliefs and vaccination intentions (Winter, Pummerer, et al., 2022). One explanation for this seeming discrepancy might lie in the fact that conspiracy belief is related to a self‐perception as being resistant to social influence but not with actual resistance (Altay et al., 2023). This makes it even more important to investigate actual social influence (ideally using experimental designs) among people with different levels of conspiracy beliefs rather than inferring resistance indirectly from correlations with other variables. This research could then also more closely investigate under which conditions, for instance, subjective norm perceptions are more (as in the case of vaccinations: Winter, Pummerer, et al., 2022) or equally strongly (as in the current work) related to the respective behaviour among individuals high (vs. low) in conspiracy beliefs.

In light of this emerging evidence on (effective) social influence among conspiracy believers, one might wonder how their increased tendency to engage in non‐normative behaviours (e.g., non‐normative collective action, non‐adherence to public health guidelines; Imhoff et al., 2021; Pummerer et al., 2025; van Mulukom et al., 2022) can be explained. It is crucial to note that the occurrence of these behaviours does not necessarily imply that this is due to their non‐normativity. Rather, they might be a consequence of the different social reality those who believe in conspiracy theories experience (e.g., the perception of others' negative intent; Frenken & Imhoff, 2022). One important aspect in this regard is that in order for social norms to take effect, they need to be perceived as such. It has been argued that conspiracy beliefs are associated with a lower perception of (especially descriptive) norms (Pummerer, 2022). In the current studies, there were no signs of a lower descriptive norm perception among individuals believing in conspiracy theories. At the same time, those believing in conspiracy theories about climate change perceived slightly lower expectations of society and important others to act pro‐environmentally themselves. Crucially, however, if they perceived high injunctive or subjective norms, they were as likely to adhere to them.

### General and specific conspiracy beliefs in the environmental domain

The current research also provides novel insights with regard to the role of different types of conspiracy beliefs for pro‐environmental behaviour. Going beyond previous work that treated conspiracist worldview as a potential moderator of the effects of social norms (Pummerer et al., 2024; Winter, Pummerer, et al., 2022), the current work investigates both general and specific conspiracy beliefs. Notably, one could have assumed that individuals who believe conspiracy theories about a specific topic (e.g., climate change) are less susceptible to social influence – compared to those who have a general conspiracist worldview, but might not (yet) believe in the context‐specific conspiracy theory (Imhoff et al., 2022). Indeed, previous work showed that the effect of information provision on pro‐environmental intentions was drastically reduced when looking at context‐specific conspiracy beliefs (rather than a conspiracist worldview) as a moderator (Winter, Hornsey, et al., 2022). Such a difference did not occur in the current studies, suggesting that even those who believe a context‐specific conspiracy theory can be influenced by social norms.

In addition, we confirmed the results of a previous meta‐analysis (Biddlestone et al., 2022) in that climate change conspiracy belief is negatively related to self‐reported pro‐environmental behaviour. For a conspiracist worldview, the pattern was less consistent and seemed to depend on the measure used. When using an average score of agreement with several popular conspiracy theories (e.g., about the Apollo moon landing or the assassination of John F. Kennedy), the bivariate correlation between conspiracist worldview and self‐reported pro‐environmental behaviour was actually positive. Given that this contradicts previous findings – which were also yielded with US samples – on climate scepticism being associated with this measure (Hornsey et al., 2018a; Lewandowsky et al., 2013), this correlation needs replication. When using a measure of conspiracy mentality (Bruder et al., 2013), there was a negative correlation between conspiracist worldview and self‐reported pro‐environmental behaviour, which was, however, considerably smaller than for climate change conspiracy beliefs. This resonates with recent work identifying conspiracy mentality as a main predictor of opposition to wind farms and agreement with misinformation about wind farms (Winter et al., 2024, 2025; Winter, Hornsey, et al., 2022). Given the inconsistency of the correlations found here and the fact that there was no correlation between conspiracy mentality and individuals' general pro‐environmental worldview in previous work (Winter et al., 2024), one might argue that a conspiracist worldview is not related to less concern for the environment or less pro‐environmental behaviour per se (for a similar reasoning, see Tam & Chan, 2023). One possibility that could account for the negative relationship we found is that a higher conspiracy mentality predisposes individuals to believe in context‐specific conspiracy theories (e.g., about climate change; Imhoff et al., 2022). This mediation hypothesis could be tested in future research.

Future work might also investigate whether conspiracy mentality (or other measures of a conspiracist worldview) relates differently to everyday pro‐environmental behaviours (such as the ones we investigated here) and behaviours that have a political component (such as protesting against wind farms). In the latter case, less pro‐environmental behaviour among those with a strong conspiracist worldview could be driven by mistrust in those public institutions that aim to accelerate a sustainable transformation of society (Pummerer, Böhm, et al., 2022).

### Limitations and future research

One obvious limitation is the cross‐sectional nature of the studies. Longitudinal studies might help to further clarify whether conspiracy beliefs temporally precede lower pro‐environmental behaviour or if maybe conspiracy theories are used to rationalize people's choice to not act pro‐environmentally (for a similar reasoning, see van Prooijen & Böhm, 2023). Similarly, social norm perceptions could be influenced by people's own pro‐environmental behaviour, since the tendency to infer the social norm from one's own behaviour (i.e., a false consensus effect) has also been found in the environmental domain (Sparkman et al., 2022). To be able to draw causal inferences, an experimental approach would be more appropriate. However, an experimental manipulation of conspiracy beliefs can raise ethical objections. Thus, one might start using experimental approaches to make prevailing social norms salient or change the perception of social norms, which has proven effective in fostering pro‐environmental attitudes and behaviours (Bergquist et al., 2019, 2023; Constantino et al., 2022; Rode et al., 2025; Unsworth & Fielding, 2014).

We observed relatively high correlations between some of the variables. This was particularly the case for the different norm perceptions but also for norms and self‐reported behaviour and especially in Study 1. It could be that participants were simply not able to meaningfully distinguish between the different sets of questions when asked to evaluate the same behaviours successively several times. To alleviate this problem, we temporally separated the assessment of predictors and outcomes in Study 3. Although this procedure runs the danger of participant drop‐out, it seems advisable in cases where measures of different concepts are otherwise hard to disentangle.

Another potential criticism pertains to our measure of pro‐environmental behaviour. We relied on a self‐report of individuals' (actual) behaviour, which is principally prone to distortions such as socially desirable responses. However, similar measures using self‐reported pro‐environmental behaviours have been shown to obtain high construct validity (Kaiser & Wilson, 2004). In addition, the valid criticism that self‐reports of pro‐environmental behaviour limit conclusiveness, generalizability and impact of the findings applies less to multi‐item scales (such as the ones we used) that ask for a variety of different pro‐environmental behaviours (Lange et al., 2023). Future research should include more diverse and consequential measures of pro‐environmental behaviour. For instance, one could strengthen the robustness and practical relevance of the current findings by replicating them with a more specific and high‐impact pro‐environmental behaviour such as the purchase of an electric vehicle – which has been shown to be positively related to social norm perceptions (Barth et al., 2016) and negatively related to conspiracy beliefs (Bretter et al., 2025).

Last but not least, it should be noted that the choice of investigated countries was not theoretically informed but rather based on feasibility considerations. Participants from the US, UK and Germany are easily reached via classical survey platforms such as MTurk or Prolific, plus we were able to provide the survey in participants' native language quite effortlessly. This choice comes with obvious limitations, because the countries are quite similar to each other in that they can be conceived as Western, educated, industrialized, rich and democratic (typically dubbed WEIRD; e.g., Henrich et al., 2010), which can severely limit the generalizability of research findings (e.g., Cheon et al., 2020). Accordingly, it would be advisable to recruit samples from broader cultural backgrounds in future studies. This might be particularly relevant when studying the interplay between social norms and individual‐level beliefs (such as conspiracy beliefs). There are studies pointing out that social norms rather than individual beliefs are particularly relevant predictors of pro‐environmental behaviour in collectivistic cultures, suggesting that norms are even more important in these contexts (e.g., Eom et al., 2016; Sherman et al., 2022). Despite these limitations, there seems to be some variation in the level of agreement with conspiracy theories and misinformation about environmental topics (Bretter et al., 2025; Ibbetson, 2021; Winter et al., 2024) as well as in the degree of polarization with regard to issues such as climate change (Kulin et al., 2021; McCright et al., 2016) in the investigated countries.

### Practical implications

From a practical viewpoint, the results can be interpreted either through an optimistic or a pessimistic lens. The pessimistic lens states that conspiracy beliefs are a potential obstacle to individual pro‐environmental behaviour and, indeed, our results show that those holding strong conspiracy beliefs start with a lower default tendency to show pro‐environmental behaviour. Optimistically, however, this lower default could be improved through changing the (perceived) expectations of important others and even larger society to act pro‐environmentally. This underscores the crucial role those social norms play in shaping pro‐environmental behaviour (Cialdini & Jacobson, 2021; Helferich et al., 2023) and allows for an optimistic outlook on norm‐based interventions targeting pro‐environmental behaviour (Cialdini et al., 2006; Hamann et al., 2015; Nolan et al., 2008) – even among those with strong conspiracy beliefs. Given that we only assessed perceived social norms in our studies, to get closer to interventions, it would be necessary to test the effects of normative feedback or information depending on individuals' conspiracy beliefs.

## CONCLUSION

The current results provide no evidence that believing in conspiracy theories (in general or about climate change in particular) comes with less adherence to pro‐environmental social norms. Contrarily, perceiving high pro‐environmental expectations of either important others (i.e., subjective norm) or larger society (i.e., injunctive norm) as well as, to some extent, perceiving that many people in society act pro‐environmentally (i.e., descriptive norm) was associated with more self‐reported pro‐environmental behaviour – irrespective of individuals’ tendency to believe in conspiracy theories. These findings replicate and extend previous work on the interplay between conspiracy beliefs and social norms. Thus, the studies might help to better understand the social reality and social factors that (do not) have an influence on people who believe in conspiracy theories. When it comes to pro‐environmental behaviour, it seems that (also) those believing in conspiracy theories are influenced by the social norms they perceive.

## AUTHOR CONTRIBUTIONS

**Kevin Winter:** Conceptualization; investigation; writing – original draft; methodology; validation; writing – review and editing; formal analysis; project administration; data curation; supervision; resources; visualization. **Lotte Pummerer:** Conceptualization; methodology; writing – review and editing; investigation; supervision. **Kai Sassenberg:** Writing – review and editing; conceptualization; resources.

## Supporting information


Appendix S1

## Data Availability

Data and code are available via PsychArchives (data: https://doi.org/10.23668/psycharchives.21209, code: https://doi.org/10.23668/psycharchives.21210).

## Footnotes

^1^
Note that the verbatim formulations differ slightly from the preregistrations in that the hypotheses formulated in the main manuscript are more inclusive (e.g., with regard to different the types of conspiracy belief) and resolve the predicted interactions differently in order to better suit the theoretical argumentation. Crucially, the focal interaction effects indicative for testing the hypotheses are statistically equivalent between the preregistration and the analyses reported in the manuscript.
