# Supplementary material for: Not that different after all: Pro‐environmental social norms predict pro‐environmental behaviour (also) among those believing in conspiracy theories
Source: Br J Psychol. 2025 Sep 15;117(Suppl 1):S176–200. doi: 10.1111/bjop.70030 (PMC13543320; doi:10.1111/bjop.70030)
Supplement: Supplementary file 1 [file BJOP-117-S176-s001.docx]

**Supplementary Information for**

**“Not that different after all: Pro-environmental social norms predict pro-environmental behavior (also) among those believing in conspiracy theories”**

List of contents

(1) Research Materials Study 1
(2) Research Materials Study 2
(3) Research Materials Study 3
(1) **Research Materials Study 1**

*Subjective norm (pro-environmental norms marked in blue)*

Please estimate how many of the people you care about say one should do the described behaviors.

**Please estimate: How many of the people you care about say one should...**

(Please answer in percent)

- React if someone cries “help”
- Not share private information with people that one doesn’t know
- Reduce the times of going shopping during the pandemic
- Ask permission to do things such as turning on the television or using the bathroom when at someone else’s home
- Use public transport in order to reduce the environmental impact
- Not talk during a movie in the cinema
- Reduce one’s meat intake for environmental reasons
- Avoid meeting people from the risk population during the pandemic
- Wear a mask in order to reduce the spread of COVID-19
- Reduce the number of direct contacts during the COVID-19 pandemic
- Reduce one’s flights in order to protect the environment
- Take the car only when necessary in order to reduce emissions
- Not lie to a friend
- Not interrupt others
- Call to let someone know if one is going to be later r not going to show up for an appointment
- Get vaccinated against COVID-19

(0; 10; 20; 30; 40; 50; 60; 70; 80; 90; 100)

*Descriptive norm of important others*

Please estimate how many of the people you care about are doing the described behaviors.

**Please estimate: How many of the people you care about...**

(Please answer in percent)

*Same items as above*

*Injunctive norm (society)*

Please estimate, how many people in society say that one should do this behavior.

**Please estimate: How many people in society say one should...**

(Please answer in percent)

*Same items as above*

*Descriptive norm (society)*

Please estimate how many people in society are doing the described behaviors.

**Please estimate: How many people in society...**

(Please answer in percent)

*Same items as above*

*Self-reported behavior (pro-environmental behaviors marked in blue)*

You will see the behaviors one last time. Now we ask you, whether you display the behaviors.

Please indicate your agreement/disagreement with the following sentences.

- I do not share private information with people that I do not know
- I take the car only when necessary in order to reduce emissions
- I ask permission to do things such as turning on the television or using the bathroom when at someone else’s home
- I use public transport in order to reduce the environmental impact
- I call to let someone know if one is going to be late or not going to show up for an appointment
- I do not interrupt other
- I am or will get vaccinated against COVID-19
- I reduce meat intake for environmental reasons
- I reduce the number of direct contacts during the COVID-19 pandemic
- I avoid meeting people from the risk population during the pandemic
- I do not lie to a friend
- I reduce flights in order to protect the environment
- I react if someone cries “help”
- I am wearing a mask in order to reduce the spread of COVID-19
- I reduce the times of going shopping during the pandemic
- I do not talk during a movie in the cinema

(1 = totally disagree; 11 = totally agree)

*Belief in conspiracy theories (climate change conspiracy theories marked in blue; items used to measure conspiracist worldview marked in orange)*

We will now ask you some questions related to your perception of events. Please read all instructions carefully and answer the questions spontaneously.

- News outlets are exaggerating numbers and danger of COVID-19
- Powerful people are using COVID-19 in order to crash the economy
- The panic about COVID-19 is partly caused by people trying to hurt the political system
- It is important to think about the economy rather than to panic about a virus that is not so dangerous after all
- COVID-19 is just one way of the government to restrict the power of the small people
- Burning fossil fuels increases atmospheric temperature (R)
- Climate change is a hoax
- Human behavior contributes to global warming (R)
- Climate change is real (R)
- The Apollo moon landings never happened and were staged in a Hollywood film studio
- The assassination of John F. Kennedy was not committed by the lone gunman Lee Harvey Oswald but was rather a detailed organized conspiracy to kill the President
- The U.S. government allowed the 9-11 attacks to take place so that it would have an excuse to achieve foreign (e.g., wars in Afghanistan and Iraq) and domestic (e.g., attacks on civil liberties) goals that had been determined prior to the attacks
- Princess Diana’s death was not an accident but rather an organized assassination by members of the British royal family who disliked her
- A powerful and secretive group known as the New World Order are planning to eventually rule the world through an autonomous world government which would replace sovereign governments
- The assassination of Martin Luther King Jr. was the result of an organized conspiracy by U.S. government agencies such as the CIA and FBI

(1 = strongly disagree; 7 = strongly agree)

(2) **Research Materials Study 2**

(original materials were in German)

*Self-reported pro-environmental behavior*

**How do you behave in your everyday life?**

Please indicate how often you conduct the following behaviors in your everyday life.

- I boycott products of companies that evidently act environmentally damaging
- I abstain from dairy products (e.g., butter, cheese, yogurt, milk)
- I take a shower instead of a bath
- I put on something warmer instead of turning up the heating, if it’s too cold in my home
- I use reusable products instead of disposable products (e.g., cloth bags instead of plastic bags, thermos flasks instead of “coffee-to-go cups”)
- I take used glass to the collection container
- I wait until I have a full drum of laundry before I wash
- I repair broken items instead of buying something new straight away
- I buy second-hand products or clothes instead of buying something new
- I abstain from meat consumption
- I hang my laundry out to dry instead of using a tumble dryer
- I buy organic instead of conventional food
- For longer trips (6 hours or longer), I take the train or bus instead of driving or flying
- For short distances, I avoid using the car and use my bike or public transport or walk instead
- I buy seasonal fruit and vegetables

(1 = never, 2 = rarely, 3 = sometimes, 4 = often, 5 = always)

*Descriptive norm*

**How do people in Germany behave in their everyday lives?**

Please estimate: How many percent of the people in Germany show the following behaviors *often* or *always*?

- boycott products of companies that evidently act environmentally damaging
- abstain from dairy products (e.g., butter, cheese, yogurt, milk)
- take a shower instead of a bath
- put on something warmer instead of turning up the heating, if it’s too cold in their homes
- use reusable products instead of disposable products (e.g., cloth bags instead of plastic bags, thermos flasks instead of “coffee-to-go cups”)
- take used glass to the collection container
- wait until I have a full drum of laundry before they wash
- repair broken items instead of buying something new straight away
- buy second-hand products or clothes instead of buying something new
- abstain from meat consumption
- hang their laundry out to dry instead of using a tumble dryer
- buy organic instead of conventional food
- take the train or bus for longer trips (6 hours or longer) instead of driving or flying
- avoid using the car and use their bike or public transport or walk instead for short distances
- buy seasonal fruit and vegetables

(0%; 100%)

*Injunctive norm*

**Now it is not about the actual behavior of others but about what they think one should do.**

Please estimate: How many percent of the people in Germany say *one should…*?

*Same items as above*

*Subjective norm*

**Now it is not about the people in Germany but about how people who are important to you personally think.**

Please estimate: How many percent of the people who are important to you say *one should…*?

*Same items as above*

*Climate change conspiracy belief*

In how far to you agree with the following statements on climate change?

- The official United Nations reports on climate change are deliberately inaccurate
- Scientists are creating panic about climate change because it is in their interests to do so
- “Climate change” is a myth promoted by the government as an excuse to raise taxes and curb people's freedom
- The “science” behind climate change is at least dubious
- The idea that the world is headed for catastrophic climate change is a fraud

(1 = do not agree at all; 7 = fully agree)

*Conspiracy mentality*

**Now we are interested in your general opinion on societal events.**

In how far do you agree with the following statements?

- Many very important things happen in the world, which the public is never informed about
- Politicians usually do not tell us the true motives for their decisions
- Government agencies closely monitor all citizens
- Events which superficially seem to lack a connection are often the result of secret activities
- There are secret organizations that greatly influence political decisions

(1 = do not agree at all; 7 = fully agree)

(3) **Research Materials Study 3**

*Subjective norm*

**How do you evaluate people who are important to you?**

We are interested in your evaluation of how **people who are important to you** think about everyday behaviors. It is not about their actual behavior but about **what they think one should do.**

Please estimate: What percentage of *the people who are important to you say one should…*?

- boycott products of companies that evidently act environmentally damaging
- abstain from dairy products (e.g., butter, cheese, yogurt, milk)
- take a shower instead of a bath
- put on something warmer instead of turning up the heating, if it’s too cold in their homes
- use reusable products instead of disposable products (e.g., cloth bags instead of plastic bags, thermos flasks instead of “coffee-to-go cups”)
- take used glass to the collection container
- wait until I have a full drum of laundry before they wash
- repair broken items instead of buying something new straight away
- buy second-hand products or clothes instead of buying something new
- abstain from meat consumption
- hang their laundry out to dry instead of using a tumble dryer
- buy organic instead of conventional food
- take the train or bus for longer trips (6 hours or longer) instead of driving or flying
- avoid using the car and use their bike or public transport or walk instead for short distances
- buy seasonal fruit and vegetables

(0%; 100%)

*Climate change conspiracy belief*

In how far to you agree with the following statements on climate change?

- Climate change is a hoax
- The official United Nations reports on climate change are deliberately inaccurate
- Scientists are creating panic about climate change because it is in their interests to do so
- The idea that the world is headed for catastrophic climate change is a fraud
- The “science” behind climate change is at least dubious
- “Climate change” is a myth promoted by the government as an excuse to raise taxes and curb people's freedom
- Climate change has been made up by climate researchers to chase funding

(1 = do not agree at all; 7 = fully agree)

*Conspiracy mentality*

**Now we are interested in your general opinion on societal and political events.**

In how far do you agree with the following statements?

- Many very important things happen in the world, which the public is never informed about
- Politicians usually do not tell us the true motives for their decisions
- Government agencies closely monitor all citizens
- Events which superficially seem to lack a connection are often the result of secret activities
- There are secret organizations that greatly influence political decisions

(1 = do not agree at all; 7 = fully agree)

*Self-reported pro-environmental behavior*

**How do you personally behave in your everyday life?**

Please indicate how often you personally show the following behaviors in your everyday life.

- I boycott products of companies that evidently act environmentally damaging
- I abstain from dairy products (e.g., butter, cheese, yogurt, milk)
- I take a shower instead of a bath
- I put on something warmer instead of turning up the heating, if it’s too cold in my home
- I use reusable products instead of disposable products (e.g., cloth bags instead of plastic bags, thermos flasks instead of “coffee-to-go cups”)
- I take used glass to the collection container
- I wait until I have a full drum of laundry before I wash
- I repair broken items instead of buying something new straight away
- I buy second-hand products or clothes instead of buying something new
- I abstain from meat consumption
- I hang my laundry out to dry instead of using a tumble dryer
- I buy organic instead of conventional food
- For longer trips (6 hours or longer), I take the train or bus instead of driving or flying
- For short distances, I avoid using the car and use my bike or public transport or walk instead
- I buy seasonal fruit and vegetables

(1 = never, 2 = rarely, 3 = sometimes, 4 = often, 5 = always)
